# Supplementary material for: Cortical and Trabecular Bone Modeling and Implications for Bone Functional Adaptation in the Mammalian Tibia
Source: Bioengineering (Basel). 2024 May 20;11(5):514. doi: 10.3390/bioengineering11050514 (PMC11118124; doi:10.3390/bioengineering11050514)
Supplement: Supplementary file 1 [file bioengineering-11-00514-s001.zip › bioengineering-3001660-supplementary.pdf]

Table S1. Parameters of mechanical stimuli that were demonstrated to influence bone modeling.

Mechanical stimuli feature	Load	Loading duration	Loading method	Animal	Sex	Bone	Age	Number	Finding	Reference
Magnitude	8.7 to 13 N	3 days/week for 2 weeks (40 cycles, 10 s rest between cycles, 2 Hz)	Axial compression	C57BL/J6 mice	F	Tibia	8, 12 and 20 weeks	At least 4 per group	Significant magnitude-dependent increases in cortical bone formation for all age groups. Significant magnitude-dependent increases in trabecular bone formation only in 8 weeks.	De Souza et al. 2005
Magnitude	8.8 to 12.4 N	3 days and then 1 day rest for 2 weeks (4 cycles x55, 3 s rest between cycles, 2 Hz)	Axial compression	C57BL/6NHsd mice	F	Tibia	8 weeks	25	Significant magnitude-dependent increases in cortical bone formation. Significant magnitude-dependent increases in trabecular bone formation.	Berman et al. 2015
Magnitude	5 to 9 N	3 days/week for 4 weeks (360 cycles, 2 Hz)	Axial compression	C57BL/J6 mice	F	Tibia	16 weeks	n = 33	Significant magnitude-dependent increases in cortical bone formation. Significant magnitude-dependent increases in trabecular bone formation only at 9N.	Weatherholt et al. 2013
Magnitude	8 or 10.5 N, 7.5 or 10 N, and 7 or 9 N, (load pairs correspond to 3 age groups respectively)	5 days/week for 2 weeks	Axial compression	C57BL/J6 mice	F	Tibia	5, 12 and 22 months	n = 12–15 per group (total of 6 groups)	Significant magnitude-dependent increases in cortical bone formation for all age groups (however, effect is less in 12- and 22-month-old mice). Significant magnitude-dependent increases in trabecular bone formation only at 5-month-old mice.	Holguin et al. 2014
Magnitude	10 or 20 N	5 days/week for 4 weeks	Lateral (side) loading	New Zealand white rabbits	?	Distal femur	31 weeks	n = 20	Increases in trabecular bone formation for both 10 and 20N (i.e., not magnitude-dependent).	Yang et al. 2013
Magnitude	25 or 30 N	3 days/week for 3 weeks	4-point bending	Sprague-Dawley rats	F	Tibia	6 months	n = 70	Significant magnitude-dependent increases in cortical bone formation.	Cullen et al. 2001

Magnitude	0 to 14 N	Days 5, 7, 9, 11, 13, 15, 17, and 19 for 3 weeks (40 cycles, 10 s rest between cycles)	Axial loading (after sciatic neurectomy)	C57BL/J6 mice	F	Tibia	17 weeks	n = 48	Significant magnitude-dependent increases in cortical bone formation. Significant load magnitude- dependent increases in trabecular bone formation.	Sugiyama et al. 2012
Magnitude	4.3 to 18 N	5 days/week for 2 weeks (360 cycles, 1, 5, or 15 Hz)	Axial compression	Sprague-Dawley rats	F	Ulna	7-8 months	n = 60	Significant magnitude-dependent increases in cortical bone formation.	Hsieh and Turner, 2001
Magnitude	1000, 1250 or 1600 $\mu\varepsilon$	3 days/week for 3 weeks (36 cycles)	Cantilever bending	C57BL/6 mice	F	Tibia	16 weeks	n = 21	Significant magnitude-dependent increases in cortical bone formation.	Srinivasan et al. 2007
Magnitude	3.5 to 7 N	1, 3 or 5 days/week for 2 weeks (4 cycles x54 with 5 s rest between cycles)	Axial compression	C57BL/J6 mice	F	Tibia	15 weeks	n = 174	Significant magnitude-dependent increases in cortical bone formation.	Yang et al. 2021
Sign	37 N	Daily for 40 days (108 cycles per day)	4-point bending (lateral surface in compression, anteromedial surface in tension)	Sprague-Dawley rats	F	Tibia	Not specified, 275 gr	n = 2	New cortical bone formation took place in both compression and tension surfaces. No significant difference in bone formation between the lateral (compression) and anteromedial (tension) surfaces.	Turner et al. 1991
Sign	---	---	Retrospective (postmortem inspection)	Rocky Mountain mule deer	M	Calcaneus	1.5 – 3 years	n = 68 (34 x2, left & right)	Larger increase in cortical bone thickness and mineralization in the compressive cortex (cranial) compared to the tensile cortex (caudal).	Skedros et al. 2001
Number of cycles	37 N	10 days out of 12 days (1, 4, 12, 36, and 108 cycles)	4-point bending (lateral surface in compression, anteromedial surface in tension)	Sprague-Dawley rats	F	Tibia	Not specified, 275 gr	n = 10	New cortical bone formation in both lateral (compression) and anteromedial (tension) surfaces. No duration-dependent increases were observed above 1 cycle per day.	Turner et al. 1991

Number of cycles	18 N	Daily for 4 weeks (10, 25 and 50 cycles)	Lateral (side) loading	New Zealand white rabbits	M	Distal femur	8-9 month	n = 14	Increases in trabecular bone formation for all durations, but not duration-dependent.	Van der Meulen 2006
Number of cycles	9 N	5 days/week for 2 weeks (4 cycles x9, 54, or 300, with 0.1 s rest between cycles, 4 Hz)	Axial compression	C57BL/6 mice	F	Tibia	16 weeks	n = 30	Significant duration-dependent increases in cortical bone formation. Significant duration-dependent increases in trabecular bone formation only between 36 and 216 cycles per day.	Yang et al. 2017
Number of cycles	25 or 30 N	3 days/week for 3 weeks (40, 120, or 400 cycles)	4-point bending	Sprague-Dawley rats	F	Tibia	6 months	n = 70	Significant duration-dependent increases in cortical bone formation at 30 N (but only from 120 to 400 cycles at 25 N).	Cullen et al. 2001
Number of cycles	1250 με	3 days/week for 3 weeks (10, 50, 250 cycles)	Cantilever bending	C57BL/6 mice	F	Tibia	16 weeks	n = 35	Increases in cortical bone formation for all durations, but not duration-dependent.	Srinivasan et al. 2007
Circadian# rhythm	11 N	5 days/week for 2 weeks (load at 8 am or 8 pm, 216 cycles, 4 Hz)	Axial compression	C57BL/6 mice	F	Tibia	10 weeks	n = 19	Greater cortical bone formation in the 8 pm group compared to the 8 am group (but only at endosteum). No circadian effect on trabecular bone formation.	Bouchard et al. 2022
Strain rate	20 N	5 days/week for 2 weeks (strain rate of 0, 0.013, 0.026, and 0.100 1/s)	Axial compression	Sprague-Dawley rats	M	Ulna	7 weeks	n = 22	Significant strain-rate dependent increases in cortical bone formation.	Mosley and Lanyon, 1998
Strain rate	54 N	Daily for 2 weeks (strain rate of 0.018, 0.030, and 0.039 1/s)	4-point bending	Sprague-Dawley rats	F	Tibia	8-9 months	n = 32	Significant strain-rate dependent increases in cortical bone formation.	Turner et al. 1995
Frequency*	4.3 to 18 N	5 days/week for 2 weeks (360 cycles, 1, 5, or 15 Hz)	Axial compression	Sprague-Dawley rats	F	Ulna	7-8 months	n = 60	Significant frequency-dependent increases in cortical bone formation.	Hsieh and Turner, 2001

Frequency*	52 N	Daily for 2 weeks (0.05, 0.1, 0.2, 0.5, 1.0, or 2.0 Hz, 36 cycles)	4-point bending	Sprague-Dawley rats	F	Tibia	8 months	n = 114	Significant frequency-dependent increases in cortical bone formation above 0.2 Hz (0.5, 1.0 and 2.0 Hz).	Turner et al. 1994
Rest	1000, 1250 or 1600 µƐ	3 days/week for 3 weeks (36 cycles, 0 or 10 s rest between cycle)	Cantilever bending	C57BL/6 mice	F	Tibia	16 weeks	n = 42	10 s rest between loading cycles did not improve cortical bone formation compared to 0 s rest.	Srinivasan et al. 2007
Rest	54 N	Days 1, 3, 5, for 2 weeks (60 x6, 90 x4, 180 x2, or 360 x1 cycles, with 2, 4, 6 or 0 h rest between cycles, respectively)	4-point bending	Sprague-Dawley rats	F	Tibia	???	n = 60	Rest-dependent increases in cortical bone formation (60 cycles x6 with 2 hours rest between each session demonstrated highest cortical bone formation.	Robling et al. 2000
Rest	54 N	5 days/week for 2 weeks (36 cycles, 0.5, 3.5, 7 or 14 s res between cycles)	4-point bending	Sprague-Dawley rats	F	Tibia	Adult	n = 60	14 s rest between loading cycles significantly increased cortical bone formation (0.5, 3.5 and 7 s did not).	Robling et al. 2001
Rest	9 N	5 days/week for 2 weeks (4 cycles x54, 0.1 or 10 s rest between cycles)	Axial compression	C57BL/6 mice	F	Tibia	16 weeks	n = 20	10 s rest between loading cycles did not improve cortical bone formation compared to 0 s rest.	Yang et al. 2017
Rest	800 µƐ	5 days/week for 3 weeks (30 Hz for 100s or 30 Hz for 1s + 10s rest for 100s)	Cantilever bending	C57BL/6 mice	F	Tibia	16 weeks	n = 18	10 s rest between loading cycles significantly improve cortical bone formation.	LaMothe and Zernicke 2004

* Frequency - number of loading cycles per second. Rest = length of rest between stimuli. Strain rate = change in strain with respect to time (e.g., running vs. walking).

# Circadian rhythm – time of day the loading takes place (number of hours after sunrise).

Table S2. Studies reporting cortical bone modeling in the murine tibia.

Animal	Sex	Age	n	Loading method	Load	Loading duration	Finding	Reference
C57BL/J6 mice	F	8, 12 and 20 weeks	At least 4 per group	Axial compression	8.7, 10, 11, 12, and 13 N	3 days/week for 2 weeks (40 cycles, 10 s rest between cycles, 2 Hz)	Exercise-induced bone formation was observed at the proximal-, mid-, and distal-shaft (highest at mid-shaft).	De Souza et al. 2005
C57BL/6NHsd mice	F	8 weeks	25	Axial compression	8.8, 10.6, and 12.4 N	3 days and then 1 day rest for 2 weeks (4 cycles x55 with 3 s rest between cycles, 2 Hz).	Exercise-induced bone formation (cortical area and thickness) primarily in the anterior-posterior direction.	Berman et al. 2015
C57BL/J6 mice	F	16 weeks	33	Axial compression	5, 7, and 9 N	3 days/week for 4 weeks (360 cycles, 2 Hz)	Exercise-induced bone formation (cortical area) in mid-diaphysis.	Weatherholt et al. 2013
C57BL/J6 mice	F	10 weeks	19	Axial compression	11 N	5 days/week for 2 weeks (216 cycles, 4 Hz)	Exercise-induced bone formation (cortical area and thickness).	Bouchard et al. 2022
C57BL/J6 mice	F	15 weeks	174	Axial compression	3.5, 5.2, and 7 N	1, 3 or 5 days/week for 2 weeks (4 cycles x54 with 5 s rest between cycles)	Exercise-induced bone formation (only significant for 5.2 and 7 N).	Yang et al. 2021
Sprague-Dawley rats	F	Not specified, 275 gr	2	4-point bending	37 N	Daily for 40 days (108 cycles per day)	Exercise-induced bone formation in both compression (lateral) and tension (anteromedial) surfaces. No significant difference in bone formation between compression and tension surfaces.	Turner et al. 1991
C57BL/6J & C3H/HeJ mice	F	16 weeks	12	4-point bending	9.3 N	6 days/week for 2 weeks (36 cycles, 2 Hz)	Exercise-induced periosteal bone formation in both mice strains (cortical area also increased but was not statistically significant).	Akhter et al., 1998
C57Bl/6J mice	F	10 weeks	15	cantilever bending	0.3 N	5 days/week for 1 week, then 3 weeks rest 1(00 cycles, 1 Hz)	Exercise-induced bone formation (cortical area and thickness).	Gross et al. 2002
BALB/c mice	M	7-8 and 21-22 months	64	Axial compression	8, 10, 12 N	5 days/week for 1 week (60 cycles, 10 s rest between cycles)	Exercise-induced bone formation (cortical area and thickness). No significant difference in new bone formation between age groups (no diminished responsiveness to loading with age).	Brodt and Silva 2010
C57Bl/6J mice	F	26 weeks	36	Axial compression	5.9 and 11.3 N	5 days/week for 2 weeks (1200 cycles, 4 Hz)	Exercise-induced bone formation (cortical area) only at 11.3 N.	Lynch et al., 2011
C57BL/6-C129/J mice	M	2 months	30	Axial compression	8 and 12 N	5 days/week for 1 week (60 cycles, 10 s rest between cycles)	Exercise-induced bone formation (cortical area) only at 12 N.	Grimston et al., 2012
BALB/c	F	2, 4, 7, and 12 months	63	Axial compression	7.5, 10.0, 8.5 and 11.0 N (loads	3 alternating days/week for 6 week (60 cycles, 10 s rest between cycles)	Exercise-induced bone formation in all age groups (cortical volume) due to periosteal maintenance and expansion.	Silva et al., 2012

					corresponding to 4 age groups respectively)			
C57BL/6 and BALB/c mice	F	4 months	49	Axial compression	10 N	3 days/week for 6 weeks (60 cycles, 10 s rest between cycles, or 1200 cycles 01.1 s rest between cycles)	Exercise-induced bone formation (cortical area and thickness) in both mice strains and loading protocols.	Holguin et al., 2013
C57Bl/6 mice	F	5, 12, and 22 months	70	Axial compression	8 or 10.5 N, 7.5 or 10 N, and 7 or 9 N, (load pairs correspond to 3 age groups respectively)	5 days/week for 2 weeks (1200 cycles, 0.1 s rest between cycles, 4 Hz)	Exercise-induced bone formation in both loading protocol (yet stronger in higher loading protocol) and 3 age groups (yet stronger at 5-month mice)	Holguin et al., 2014
C57Bl/6 mice	F	6, 10, and 16 weeks	18	Axial compression	9.1, 9.1, and 8.8 N (loads correspond to 3 age groups respectively)	5 days/week for 2 weeks (1200 cycles, 0.1 s rest between cycles, 4 Hz)	Exercise-induced bone formation (cortical area), yet the exercise-induced response decreased with age.	Main et al., 2014
C57Bl/6 mice	F	16 weeks	40	Axial compression	9 N	5 days/week for 2 weeks. (4 cycles x9, 54, or 300, with 0.1 s rest between cycles, or 4 cycles x54 with 10 s rest between cycles, 4 Hz)	Exercise-induced bone formation (cortical area and thickness). however incremental increase in bone formation was reduced as cycle number increased (i.e. reduced benefit for higher cycle number).	Yang et al., 2017
C57Bl/6 mice	F	16 weeks	33	Axial compression	7 N	Group 1: 5 days/week for 2 weeks. (4 cycles x54 with 5 s rest between cycles, 4 Hz). Group 2: 3 weeks tail suspension (hindlimb unloaded)	Exercise-induced bone formation or disuse-induced bone resorption (cortical area).	Yang et al., 2019
Sprague-Dawley rats	F	13 months	35	Axial compression	Treadmill running	5 days/week for 9 or 16 weeks (gradual increase to 17 m/min for 1 h at the end of week 2 and then maintain).	Exercise-induced bone formation (cortical area and bone mineral density), yet some parameters were only statistically significant after 16 weeks of exercise.	Chen et al., 1994
C57Bl/6J mice	F	10 and 26 weeks	22	Axial compression	11 N	5 days/week for 2 weeks (4 x54 cycles, 5 sec rest between, 4 Hz)	Exercise-induced bone formation (cortical area and thickness) in both age groups (10 and 26 weeks).	Willie et al. 2013

Table S3. Studies reporting cortical bone modeling in large mammal's tibia.

Animal	Sex	Age	n	Loading method	Load	Loading duration	Finding	Reference
Dorset sheep	M	40, 265-75, or 400-30 days	37	Axial compression	Treadmill running	60 min daily for 90 days at about 4 km/h (around 6,000 loading cycles per day)	No Exercise-induced bone formation at the articular surface. Some Exercise-induced bone formation at mid-diaphysis, mostly evident in juveniles.	Lieberman et al., 2001
Dorset sheep	M	40, 265, or 415 days	36	Axial compression	Treadmill running	60 min daily for 90 days at about 4 km/h (around 6,000 loading cycles per day)	Exercise-induced periosteal bone formation, increase was not statistically significant in the adult group (415 days).	Lieberman et al., 2003
Dorset sheep	M	40 days	10	Axial compression	Treadmill running	30 min daily for 90 days at about 4 km/h (around 6,000 loading cycles per day)	Exercise-induced periosteal bone formation, but not linked to local strain magnitude during peak loading.	Wallace et al., 2014
Reindeer	M/F	unknown	122	Axial compression	Working, zoo or free ranging/wild reindeer	Retrospective study. Free ranging/wild reindeer served as reference and were compared to working (racing) and zoo (sedentary) reindeer.	Tendency for increased robusticity of long bones (cortical area), especially the tibia, in working male and zoo female reindeer, may indicate exercise-induced bone formation	Niinimäki et al., 2021

Table S4. Studies reporting trabecular bone modeling in the murine proximal tibia (unless distal tibia is indicated).

**Animal**	**Sex**	**Age**	**n**	**Loading method**	**Load**	**Loading duration**	**scan resolution (μm)**	**Finding**	**Reference**
C57BL/J6 mice	F	8, 12 and 20 weeks	At least 4 per group	Axial compression	8.7, 10, 11, 12, and 13 N	3 days/week for 2 weeks (40 cycles, 10 s rest between cycles, 2 Hz)	9.0	Exercise-induced trabecular BV/TV increase in 8w via an increase in Tb.N. Exercise-induced trabecular BV/TV decrease in 12 and 20w via an decrease in Tb.N, yet Tb.Th increased (i.e., trabecular architecture was modified).	De Souza et al. 2005
C57BL/6NHsd mice	F	8 weeks	25	Axial compression	8.8, 10.6, and 12.4 N	3 days and then 1 day rest for 2 weeks (4 cycles x55 with 3 s rest between cycles, 2 Hz)	10.2	Exercise-induced trabecular BV/TV increase in all load groups (yet only statistically significant at 12.4 N) via an increase in Tb.Th, while Tb.N decreased (i.e., trabecular architecture was modified).	Berman et al. 2015
C57BL/J6 mice	F	16 weeks	33	Axial compression	5, 7, and 9 N	3 days/week for 4 weeks (360 cycles, 2 Hz)	13.0	Exercise-induced trabecular BV/TV increase via an increase in Tb.Th only in the 9 N group. No change in Tb.N in any of the groups.	Weatherholt et al. 2013
C57BL/J6 mice	F	10 weeks	19	Axial compression	11 N	5 days/week for 2 weeks (216 cycles, 4 Hz)	8.0	Exercise-induced trabecular BV/TV increase via an increase in Tb.Th and Tb.N.	Bouchard et al. 2022
C57BL/J6 mice	F	15 weeks	174	Axial compression	3.5, 5.2, and 7 N	1, 3 or 5 days/week for 2 weeks (4 cycles x54 with 5 s rest between cycles)	10.0	Exercise-induced trabecular BV/TV increase via an increase in Tb.Th only in the 7 N group.	Yang et al. 2021
BALB/c mice	M	7-8 and 21-22 months	64	Axial compression	8, 10, 12 N	5 days/week for 1 week (60 cycles, 10 s rest between cycles)	16.0	Exercise-induced trabecular bone formation in both groups (higher mineralized surfaces). Exercise-induced trabecular BV/TV decrease via a decrease in Tb.Th in 7 month mice. A trend of exercise-induced trabecular BV/TV increase (non-significant) via an increase in Tb.Th in 22 month mice.	Brodt and Silva 2010
C57Bl/6J mice	F	26 weeks	36	Axial compression	5.9 and 11.3 N	5 days/week for 2 weeks (1200 cycles, 4 Hz)	15.0	Exercise-induced trabecular BV/TV increase via an increase in Tb.Th only in the 11.3 N group. Trabecular tissue mineral density also increased.	Lynch et al., 2011
C57BL/6-C129/J mice	M	2 months	30	Axial compression	8 and 12 N	5 days/week for 1 week (60 cycles, 10 s rest between cycles)	21.5	No exercise-induced trabecular bone formation in either load. Some indication for exercise-induced trabecular bone resorption at 12 N.	Grimston et al., 2012
C57BL/6 and BALB/c mice	F	4 months	49	Axial compression	10 N	3 days/week for 6 weeks (60 cycles, 10 s rest between cycles, or 1200 cycles 01.1 s rest between cycles)	21.0	Exercise-induced trabecular BV/TV increase via an increase in Tb.Th for both mice strains, and Tb.N for BALB/c mice, at 1200 cycles. Exercise-induced trabecular BV/TV increase via an increase in Tb.Th for both	Holguin et al., 2013

								mice strains at 60 cycles (BV/TV increase is not statistically significant in C57BL/6 mice). Exercise-induced trabecular BMD increase for both mice strains, yet difference is only not statistically significant in BALB/c mice).	
C57Bl/6 mice	F	5, 12, and 22 months	70	Axial compression	8 or 10.5 N, 7.5 or 10 N, and 7 or 9 N, (load pairs correspond to 3 age groups respectively)	5 days/week for 2 weeks (1200 cycles, 0.1 s rest between cycles, 4 Hz)	21.0	Exercise-induced trabecular BV/TV increase via an increase in Tb.Th for 5 months mice (statistically significant BV/TV increase at 10.5 N but only a trend at 8 N). Exercise-induced Tb.Th increase in 12 months mice only at 10 N. No exercise-induced trabecular BV/TV or Tb.Th increase in 22 month mice for both 7 and 9 N loads.	Holguin et al., 2014
C57Bl/6 mice	F	6, 10, and 16 weeks	18	Axial compression	9.1, 9.1, and 8.8 N (loads correspond to 6, 10, and 16 weeks respectively)	5 days/week for 2 weeks (1200 cycles, 0.1 s rest between cycles, 4 Hz)	15.0	Exercise-induced trabecular BV/TV increase via an increase in Tb.Th, and exercise-induced trabecular bone TMD increase, for all age groups. Yet exercise-induced response decreased with age.	Main et al., 2014
C57Bl/6 mice	F	16 weeks	40	Axial compression	9 N	5 days/week for 2 weeks. (4 cycles x9, 54, or 300, with 0.1 s rest between cycles, or 4 cycles x54 with 10 s rest between cycles, 4 Hz)	10.0	Exercise-induced trabecular BV/TV increase (some increases were not statistically significant) via an increase in Tb.Th (no change in Tb.N), and exercise-induced trabecular bone TMD increase.	Yang et al., 2017
C57Bl/6 mice	F	16 weeks	33	Axial compression	7 N	Group 1: 5 days/week for 2 weeks. (4 cycles x54 with 5 s rest between cycles, 4 Hz). Group 2: 3 weeks tail suspension (hindlimb unloaded)	10.0	Exercise-induced trabecular BV/TV increase via an increase in Tb.Th, while Tb.N did not change. Disuse-induced BV/TV decrease via a decrease in Tb.Th and Tb.N.	Yang et al., 2019
Sprague-Dawley rats	F	4 weeks	40	Axial compression	Treadmill running	5 days/week for 8 or 12 weeks. Speed and duration were increased gradually until end of week 3 to 24 meters/minute for 60 minutes.	Histology, proximal and distal tibia	Exercise-induced trabecular BV/TV increase in both proximal and distal tibia for rats exercised 8 and 12 weeks (trabecular BV/TV increase in the distal tibia after 8 weeks was not statistically significant). Trabecular bone formation rate increase is due to increase in mineral apposition rate.	Iwamoto et al., 1999
C57BL/6J Mice	M	10 weeks	20	Axial compression	3 N	5 days/week for 2 or 6 weeks (1200 cycles, 4 Hz)	11.5, Proximal tibia	Exercise-induced trabecular BV/TV increase via an increase in Tb.Th in both 2 and 6 weeks exercise regimes (Tb.Th increase after 2 weeks was not statistically significant). No change in Tb.N is inferred from no change in Tb.Sp.	Fritton et al., 2005

C57BL/6J mice	M	10 weeks	11	Axial compression	4.6 N	5 days/week for 6 weeks. (4 cycles x300, with 0.1 s rest between cycles, 4 Hz)	11.6, proximal tibia	Exercise-induced trabecular BV/TV increase via an increase in Tb.Th, but no change in Tb.N.	Fritton et al. 2008
Sprague-Dawley rats	M	6 months	42	Axial compression	High and low load jumps	3 days/week (alternating days) for 5 weeks. high load group gradually increased exercise to 16 jumps per session with a load of 410 g, low load group had the same number of jumps per session with a load of 30 g.	Histology, Proximal tibia	Exercise-induced trabecular BV/TV increase via an increase in Tb.Th (only a trend in the high load group) but no change in Tb.N, and exercise-induced trabecular BMD increase.	Swift et al., 2010
C57Bl/6J mice	F	10 and 26 weeks	22	Axial compression	11 N	5 days/week for 2 weeks (4 x54 cycles, 5 sec rest between, 4 Hz)	10.5, proximal tibia	Exercise-induced trabecular BV/TV increase via an increase in Tb.Th only in 10 week mice (but not in 26 week mice). No change in Tb.N.	Willie et al. 2013

BV/TV – bone volume fraction (ratio between bone volume and total volume).
Tb.Th – trabecular thickness.
Tb.N – Trabecular number.
Tb.Sp – trabecular spacing.
BMD – bone mineral density.
TMD – tissue mineral density.

Table S5. Studies reporting trabecular bone modeling in large mammal's tibia.

Animal	Sex	Age	n	Loading method	Load	Loading duration	scan resolution (µm)	Finding	Reference
Cheetahs, leopards, jaguars, and cougars	M/F	?	16, 14, 9, 14, respectively	Axial compression	Normal locomotion	Estimated daily travel distance (km/day): 4.0, 6.8, 11, and 7.4, respectively. Estimated home range size (km^2): 50-130, 8-63, 15-54 and 250, respectively.	pQCT, 100, distal tibia	Differences in home range size and estimated daily travel distance did not corelate to distal tibia BV/TV (study also looked at femora and humeri with same result). Thus, no evidence for functional-induced trabecular BV/TV adaptation.	Assif and Chirchir, 2024
Japanese macaque	M	adults	Bipedal locomotion (n = 1). Wild individuals (n = 5)	Axial compression	Bipedal locomotion	30–60 min/day from age 2 until death at 10 years	45 – 59, proximal tibia	Differences in locomotion (trained bipedal vs. wild quadrupedal) did not clearly corelate to distal tibia trabecular bone parameters (e.g., BV/TV, Tb.Th, DA etc.) The study also looked at femora from the same individuals with similar outcome. Thus, no evidence for functional-induced trabecular BV/TV adaptation.	Cazenave et al. 2023
Pig	F	6, 23, 56, 104, and 230 weeks	10 (2 per age group)	Axial compression	Normal locomotion	Retrospective study – physiological loading. Pigs were growing on a farm till they were euthanized	28, proximal tibia	At a young age, trabecular bone responds to physiological loading by increasing BV/TV. However, later, the response changes to an increase in DA (probably resorption of misaligned and minimally loaded trabeculae).	Tanck et al., 2001
Dorset sheep	M/F	4 weeks	20	Axial compression	Treadmill trotting	15 min/day, at a speed of 1.35–1.5 m/s, 6 days/week for 34 days. Group 1 (n = 8) trotted on a level treadmill. Group 2 (n = 8) trotted on an inclined treadmill (7°) and wore platform shoes for the rest of the	25-28.6, distal tibia	Level vs. incline groups demonstrated a difference of 3.6° in tarsal joint angle during peak ground reaction force. correspondingly, principal trabecular orientation in the distal tibia adjusted and was more obtuse by 2.7° to 4.3° in the incline group. Exercise-induced	Barak et al., 2011

						time to keep the same joint angle.		trabecular BV/TV increase (incline and level groups vs. sedentary group) via an increase in Tb.Th and Tb.N, but no change in DA in the distal radius.	

BV/TV – bone volume fraction (ratio between bone volume and total volume).
Tb.Th – trabecular thickness.
Tb.N – Trabecular number.
DA – degree of anisotropy.

Table S6. Cortical and trabecular bone modeling differences and similarities in mice, as reported in the literature.

Cortical bone	Trabecular bone	Reference
Exercise-induced cortical bone formation (cortical area), yet only statistically significant at higher loads (12 N but not 8 N).	No exercise-induced trabecular bone formation in either load (8 or 12 N). Some indication for unexpected exercise-induced trabecular bone resorption at 12 N.	Grimston et al. 2012
Exercise-induced cortical bone formation in all age groups (2, 4, 7, and 12 months), due to increase in cortical volume via periosteal maintenance and expansion.	Exercise-induced trabecular BV/TV decrease in all age groups (except for 2 months) mostly due to a decrease in Tb.N.	Silva et al., 2012
Exercise-induced cortical bone formation (cortical area and thickness) for all loads (8.8, 10.6, and 12.4 N).	Exercise-induced trabecular BV/TV increase in all load, yet only statistically significant at 12.4 N. BV/TV increased via an increase in Tb.Th, while Tb.N decreased.	Berman et al., 2015
Exercise-induced cortical bone formation but only statistically significant for 5.2 and 7 N (not for 3.5 N).	Exercise-induced trabecular BV/TV increase via an increase in Tb.Th only at 7 N (not for 5.2 and 3.5 N).	Yang et al., 2021
Exercise-induced bone formation was observed in all age groups (8, 12 and 20 weeks), at the proximal-, mid-, and distal-shaft (highest at mid-shaft).	Exercise-induced trabecular BV/TV increase in 8w via an increase in Tb.N. Exercise-induced trabecular BV/TV decrease in 12 and 20w via an decrease in Tb.N, yet Tb.Th increased (i.e., trabecular architecture was modified).	De Souza et al. 2005
Exercise-induced bone formation in both lower and higher magnitude loading protocol (yet stronger in higher loading protocol) and 3 age groups (5, 12, and 22 months; yet stronger at 5-month mice)	Exercise-induced trabecular BV/TV increase via an increase in Tb.Th for 5 months mice (but only statistically significant BV/TV increase in the higher magnitude loading protocol). Exercise-induced Tb.Th increase in 12 months mice only in the higher magnitude loading protocol. No exercise-induced trabecular BV/TV or Tb.Th increase in 22 month mice.	Holguin et al., 2014
Exercise-induced bone formation (cortical area) in mid-diaphysis for all 3 magnitude loading protocols (5, 7, and 9 N).	Exercise-induced trabecular BV/TV increase via an increase in Tb.Th only in the higher magnitude loading protocol. No change in Tb.N in any of the groups.	Weatherholt et al. 2013
Exercise-induced bone formation (cortical area and thickness) in both age groups (10 and 26 weeks).	Exercise-induced trabecular BV/TV increase via an increase in Tb.Th only in 10 week mice (but not in 26 week mice). No change in Tb.N.	Willie et al. 2013
Exercise-induced bone formation (cortical area). Disuse-induced bone resorption (cortical area).	Exercise-induced trabecular BV/TV increase via an increase in Tb.Th, while Tb.N did not change. Disuse-induced BV/TV decrease via a decrease in Tb.Th and Tb.N.	Yang et al., 2019
Exercise-induced cortical bone formation (cortical area and thickness) in both mice strains and loading protocols.	At 1200 cycles: Exercise-induced trabecular BV/TV increase via an increase in Tb.Th for both mice strains, and Tb.N for BALB/c mice.<br>At 60 cycles with 10 s rest: Exercise-induced trabecular BV/TV increase via an increase in Tb.Th for both mice strains (yet BV/TV increase is not statistically significant in C57BL/6 mice).	Holguin et al., 2013
Exercise-induced cortical bone formation (cortical area), for all age groups (6, 10, and 16 weeks). Yet exercise-induced response decreased with age.	Exercise-induced trabecular BV/TV increase via an increase in Tb.Th, and exercise-induced trabecular bone TMD increase, for all age groups. Yet exercise-induced response decreased with age.	Main et al. 2014
Exercise-induced cortical bone formation (cortical area and thickness). however incremental increase in bone formation was reduced as cycle number increased (36, 216 and 1200; i.e., reduced benefit for higher cycle number).	Exercise-induced trabecular BV/TV increase (difference was not always statistically significant) via a statistically significant increase in Tb.Th, while Tb.N did not change. In addition, exercise-induced trabecular bone TMD increase.	Yang et al., 2017
Exercise-induced bone formation (cortical area and thickness).	Exercise-induced trabecular BV/TV increase via an increase in Tb.Th and Tb.N. In addition, exercise-induced trabecular bone TMD increase.	Bouchard et al. 2022

Exercise-induced bone formation (cortical area) only in the higher magnitude loading protocol (11.3 N, but not 5.9 N).	Exercise-induced trabecular BV/TV increase via an increase in Tb.Th only in the higher magnitude loading protocol. Trabecular tissue mineral density also increased.	Lynch et al., 2011
Significant magnitude-dependent increases in cortical bone formation (cortical area and thickness) between 8 and 14 N.	Exercise-induced trabecular BV/TV increase via an increase in Tb.Th and\d Tb.N between 8 and 14 N.	Sugiyama et al. 2012

Note – for information about the study parameters (e.g., species, sex, age, n etc.) see tables S1-S5.

BV/TV – bone volume fraction (ratio between bone volume and total volume).
Tb.Th – trabecular thickness.
Tb.N – Trabecular number.
TMD – tissue mineral density.
